# O015. Evaluation of the genetic polymorphism of the α3 (CHRNA3) and α5 (CHRNA5) nicotinic receptor subunits, in patients with cluster headache

**DOI:** 10.1186/1129-2377-16-S1-A88

**Published:** 2015-09-28

**Authors:** Maria Michela Cainazzo, Ilaria Tiraferri, Michela Ciccarese, Angela Martinelli, Cinzia Cameli, Elena Bacchelli, Michele Zoli, Luigi Alberto Pini

**Affiliations:** Headache and Drug Abuse Inter-Department Research Centre, University of Modena and Reggio Emilia, Modena, Italy; Department of Pharmacy and Biotechnology, University of Bologna, Bologna, Italy; Department of Biomedical, Metabolic and Neural Sciences, University of Modena and Reggio Emilia, Modena, Italy

## Introduction

About 80% of patients with cluster headache (CH) have a history of cigarette smoking[1]; a common genetic basis between CH and smoking has been suggested by the identification of a gene cluster on chromosome 15q25, encoding for neuronal acetylcholine receptor subunits α3, α5 and β4 (CHRNA5-CHRNA3-CHRNB4). Receptors containing the α5 subunit contribute to nicotine withdrawal symptoms and anxiety modulation[2, 3].

## Aim

To identify rare variants with a possible role in the etiology of CH and nicotine addiction, we investigated the genetic variants into the locus CHRNA5-CHRNA3 using the blood of CH patients and compared it with the blood of control patients (case-control association study).

## Materials and methods

We enrolled 65 patients with CH, of which 53 men and 12 women; male to female ratio=4.4:1. In the sample there were 48 active smokers, 12 former smokers and 5 patients whom had never smoked. CH patients were, respectively, divided into two groups: 54 with episodic and 11 with chronic form. We analyzed three single nucleotide polymorphisms (SNPs) known to be associated with nicotine addiction (rs16969968 and rs6495306 localized on CHRNA5 gene; rs578776 localized on CHRNA3 gene) in CH patients and in a control group consisting of 263 individuals that were comparable for age, smoking status and geographic origin. The analysis of rare variants of the genes was performed by sequencing of the coding portion of the gene and 5'-untranslated region (5'UTR) with the Sanger method. The sequence and genomic organization were obtained from the University of California Santa Cruz (UCSC) genome browser (http://genome.ucsc.edu/). PLINK (http://pngu.mgh.harvard.edu/purcell/plink/) was used for the statistical analysis of the data.

## Results

The analysis of the sequences did not evidence new mutations with a functional effect on the development of disease. However, as regards the three polymorphisms selected, the comparison of the allelic frequencies in CH patients and in healthy smokers, highlighted a slight but statistically significant with regards to the SNP rs578776 localized on 3'-untranslated region (3'-UTR). The A allele, protective in the risk of developing nicotine addiction and obtained by the replacement of the aspartic acid with asparagine in position 398, is less expressed (p = 0.038) in CH patients.

## Discussion

CH patients seem to have a stronger genetic predisposition to develop smoke dependence. Probably, the excessive intake of nicotine could be associated with an up-regulation of pineal nicotinic receptor α3β4[4], and this could trigger a dysfunction of melatonin release linked to the CH's chrono-biological profile.

Written informed consent to publication was obtained from the patient(s).
